# Inhibition of cytotoxic fibril formation of α-synuclein and human insulin by Silymarin from the *Silybum marianum*

**DOI:** 10.1371/journal.pone.0320283

**Published:** 2025-05-02

**Authors:** Beitollah Moosakhani, Mahshid Taleb, Zahra Mahmoudi Eshkaftaki, Nasser Nikfarjam, Azam Serajian, Mohammad Bagher Shahsavani, Ali Akbar Meratan

**Affiliations:** 1 Department of Biological Sciences, Institute for Advanced Studies in Basic Sciences (IASBS), Zanjan , Iran; 2 Department of Chemistry, Institute for Advanced Studies in Basic Sciences (IASBS), Zanjan , Iran; 3 Protein Chemistry Laboratory (PCL), Department of Biology, College of Sciences, Shiraz University, Shiraz, Iran; Tokyo Women's Medical University, JAPAN

## Abstract

Silymarin (SIL), the extract obtained from the seeds of milk thistle (*Silybum marianum*), contains several flavonolignans with a broad range of therapeutic properties such as antioxidant, anti-inflammatory, and neuroprotective effects. Despite several studies indicating the neuroprotective effects of SIL in relating to neurodegenerative diseases (NDs), there is no report regarding the anti-amyloidogenic activity and the mechanism of action of SIL *in vitro*. Here, we have extracted SIL from the seeds of milk thistle (SIL A), followed by investigating its potential, in comparison with SIL purchased from Sigma company (SIL B), in modulating fibrillogenesis and cytotoxicity of human insulin and α-synuclein (α-syn) amyloid fibrils. The obtained results indicated the potency of both SIL A and SIL B in inhibiting the assembly process and related cytotoxicity of both proteins but via different mechanisms, including inhibition of amyloid fibrillation with the appearance of short fibrils for human insulin and redirecting the assembly process of α-syn toward the formation of small globular structures. The higher inhibitory effects of SIL B may be attributed to its higher silybin content, which is responsible for the most biological, including anti-amyloidogenic, activities of SIL B. Nanonization increased the capacity of both SILs to inhibit fibrillation and related cytotoxicity of both proteins. Taken together, these results may suggest SIL A as a potent candidate relating to NDs and highlight nanonization as a promising approach to increase its anti-amyloidogenic properties.

## Introduction

Neurodegenerative diseases (NDs), including Alzheimer’s and Parkinson’s diseases, and type II diabetes, are characterized by the pathological hallmarks of intracellular and extracellular proteinaceous deposits known as amyloid fibrils [1–3]. This suggests that the molecular basis of these devastating diseases may be traced back to the harmful effects of peptides and proteins with incorrect/toxic structures. The toxicity associated with protein aggregates may manifest in biological membranes damage and permeabilization, high levels of oxidative stress, organelle dysfunction, and proteostasis impairment, eventually leading to cell death [4–6]. The well-documented observations that preventing fibril formation can result in several cytoprotective effects have led to many efforts to develop strategies for targeting protein misfolding and amyloid fibril formation [reviewed in 7]. One of the common strategies is to inhibit amyloid fibril formation by (i) stabilizing the native form of peptides/proteins, (ii) inhibiting the formation of toxic amyloidogenic species, (iii) remodeling of toxic oligomers into non-toxic off-pathway species, and (iv) clearance of insoluble large protein aggregates. Among various compounds screened to identify anti-aggregation agents, naturally-occurring small molecules, specifically polyphenols found in vegetables, fruits, and medicinal plants, are unique and have received remarkable interest [8–10]. Moreover, the results obtained by extensive epidemiological studies indicate the reduced incidence of age-related diseases, including NDs, in diets containing high intake of flavonoids and polyphenols [11,12]. Since many age-related disorders and NDs are triggered by elevated oxidative stress [13,14], natural polyphenols with high antioxidative properties are of great interest.

Silymarin (SIL) is an extract from the seeds of milk thistle (*Silybum marianum*) that contains approximately 65% to 80% flavonolignans (silybin (silibinin), isosilybin, silychristin, silydianin, and taxifolin), with small amounts of flavonoids, and approximately 20% to 35% of fatty acids and other polyphenolic compounds [15]. SIL has been used for centuries as a natural remedy for the treatment of various diseases, including liver disorders such as chronic hepatitis and cirrhosis [16] and different types of cancers [17]. Moreover, due to its antioxidant and anti-inflammatory effects on the central nervous system (CNS) and the ability to cross the blood-brain barrier [18], SIL has gained prominence as a neuroprotective compound against CNS disorders [19], including NDs [20]. In this regard, the potential neuroprotective effects of SIL have been reported in several cellular and animal models of NDs, including Alzheimer’s [21–24] and Parkinson’s [25–28] diseases. Despite these extensive studies, there is no report directly investigate the capacity of SIL in preventing amyloid fibril formation of various disease-related and -unrelated peptides/proteins and its possible mechanism of action *in vitro*, and most performed studies are related to its active components, in particularly silybin [29–34], purchased from Sigma company.

In the present study, SIL was extracted from the seeds of *Silybum marianum*, followed by Liquid Chromatography-Mass Spectrometry (LC-MS) and High-Performance Liquid Chromatography (HPLC) experiments to confirm the correct extraction and to analysis the components of SIL. Then, the potency of extracted SIL (SIL A), in comparison with SIL purchased from Sigma company (SIL B), in modulating the assembly process of ordered human insulin and intrinsically disordered α-synuclein (α-syn) proteins was examined by a broad range of techniques. Considering our previous reports indicating the improving effect of nanonization on anti-amyloidogenic and neuroprotective effects of natural polyphenols [35–39], the impact of nanonization on the anti-amyloidogenic properties of SIL A and SIL B was investigated. We found that both SIL A and SIL B are potent inhibitors of protein aggregation and that nanonization can improve the capacity of these compounds in inhibiting amyloid fibrillation and cytotoxicity associated with human insulin and α-syn proteins. However, the mechanism by which SIL A/SIL B, and their respective nanoparticles, modulate the assembly process of two proteins may be different. This is the first report regarding the anti-amyloidogenic effects and possible mechanism of action of natural SIL (SIL A), suggesting that this compound has the prospect of further development as an efficient amyloid fibril formation inhibitor.

## Materials and methods

### SIL extraction

The seeds of *Silybum marianum* were collected from Hamedan, Iran. To increase the efficiency of SIL extraction, seeds were powdered using a grinder. Then, 100 grams of obtained powder was subjected to Soxhlet and their oil/fatty acid content was extracted using n-hexane (500 mL) overnight at room temperature, while n-hexane was replaced after 10 h. The SIL content of obtained powder was extracted by Soxhlet using methanol 80% (v/v) for 24 h [40]. Finally, the suspension was concentrated and dried in an oven at 50 °C, and the resultant brown powder, corresponding to SIL A (Fig A in S1 File), was kept in a dark place at -20 °C for subsequent experiments.

### Preparation of SIL nanoparticles

Nanonization of SIL A and SIL B was performed using direct oxidative pyrolysis according to our previous reports [36,37]. For SIL A, 500 mg of dried powder was heated at 180 °C for 10 min in an oil bath. Due to their nano-scale size, nanoparticles can scatter light and lead to the increased turbidity of solution that manifests as a color change from light to dark. As shown in Fig A in S1 File, heating resulted in a color change from brown to black indicating the synthesis of SIL A nanoparticles. To exclude un-reacted polyphenols and other compounds, which may trap between nanosheets, 100 mL NaOH (250 mM) was added and the solution was stirred for 24 h at room temperature. In fact, by deprotonating functional groups of polyphenols, including carboxylic acid and phenol, NaOH will increase the surface negative charge of nanosheets, leading to detachment of nanosheets and release of un-reacted polyphenols and other impurities that will be removed by dialysis in next steps. To remove very large particles and to obtain a homogenous solution of SIL A nanoparticles (Nano A), the suspension was filtered and dialyzed against deionized water (DW) using a cut-off 12 KDa dialysis tube. In contrast to SIL A containing several compounds with various functional groups, SIL B constituents only few compounds, mainly silybin, which may decrease the yield of nanoparticle formation. Thus, for the synthesis of Nano B, 1 gram of dried powder of SIL B (kept at -20 °C) was mixed with 1 gram of citric acid (CA), where CA acts as a cross-linking agent to increase the efficiency of nanoparticle production [41]. Like the procedure mentioned above for Nano A, the mixed powder was heated at 180 °C for 10 min, followed by neutralizing with NaOH solution and dialysis against DW. A color change from light yellow to brown indicates the synthesis of SIL B nanoparticles (Nano B) (Fig B in S1 File). Finally, the obtained powders, corresponding to Nano A and Nano B, were ground finely and kept in a dark place at -20 °C until use.

### Liquid Chromatography-Mass Spectrometry (LC-MS) analysis

The constituents of SIL A were analyzed using Shimadzu LC-MS 2010 A, equipped with an Eclipse Atlantis T3 column (100 mm × 2.1 mm × 3 µm particle size) (Waters, USA) with an electrospray ionization ESI source. The separation of SIL A components was performed according to the procedure described by Mamashli et al. [35]. Briefly, 5 µL of SIL A (10 μg/mL) was injected into the column and elution was conducted using a binary gradient of formic acid/methanol (0.1% v/v:70%) and formic acid/H_2_O (0.1% v/v:30%) at a constant flow rate of 0.2 mL/min. The column was run at room temperature and the detection gain was 1.8 kV. Probe and CDL voltages were 3.5 kV and 20 V, respectively. Grade 5 nitrogen gas was employed as the nebulizer with a flow rate of 1.2 L/min. The CDL and block temperatures were both 250 °C. The data was collected by the Lab Solutions TM software.

### HPLC analysis

The SIL A and SIL B components were determined by HPLC (Agilent, USA), according to Kvasnička [42]. Ten μL of samples (20 μg/mL) were applied onto a Nucleosil C18 column equilibrated with a degassed solution containing 85% phosphoric acid-methanol-water (0.5:46:64, v/v), as mobile phase. The elution was conducted in an isocratic mode at a flow rate of 0.8 mL/min, and to characterize SIL compounds chromatograms were recorded at 288 nm [42–44]. All measurements were performed at room temperature.

### Characterization of SILs and SILs nanoparticles

As an indicative of nanoparticle formation, the UV-Vis absorbance spectra of SIL A and SIL B and their respective nanoparticles (20 μg/mL) were recorded in the range of 200–700 nm. To examine the fluorescence properties of SIL A and SIL B, as well as Nano A and Nano B, aliquots of aqueous solution of samples (10 μg/mL) were excited in a wide range of wavelengths and their emission spectra were recorded. A slit width of 5 nm was used for both excitation and emission. Atomic force microscopy (AFM) and high-resolution transmission electron microscopy (HR-TEM) were employed to analysis the morphology of nanoparticles. For AFM, 10 μL of samples (10 μg/mL) were placed on a mica and images were acquired using a quantitative AFM in non-contact mode. For HR-TEM imaging, aliquots of nanoparticles (10 μg/mL) were placed on a carbon-coated copper grid and dried at room temperature. The images were recorded at an accelerating voltage of 200 kV using a FEI Tecnai G2 F20 SuperTwin HR-TEM.

### DPPH radical scavenging activity

A DPPH-based assay was used to determine the free radical scavenging capacity of compounds [45]. Briefly, 25 μL of SIL A, SIL B, Nano A, and Nano B solutions with various concentrations ranging from 50 to 500 μg/mL, or DW as control, was added to 475 μL of 25 μM DPPH solution dissolved in methanol. The final concentrations of compounds were in the range of 2.5–25 μg/mL. The solutions were incubated for 30 min at room temperature and then transferred into a 96-well plate. The absorbance of samples was recorded at 517 nm using a microplate reader. Ascorbic acid at a final concentration of 25 μg/mL was used as a positive control for the maximum antioxidant effect. All experiments were performed in triplicate. The antioxidant activity of compounds is expressed as the fraction of maximum effect (ascorbic acid): [(measured signal−blank signal)/(maximum signal−blank signal)] x 100.

### Sample preparation and amyloid fibril formation

Human insulin solution was prepared in 50 mM glycine buffer (pH 2.2) in a final concentration of 1.5 mg/mL (∼200 μM) [38]. Expression of recombinant human α-syn was performed on Escherichia coli BL21 containing plasmid pT7- 7 (Addgene) and the expressed protein was purified as described previously [46]. Since methanol 80% (v/v) was used for the extraction of SIL from the seeds of *Silybum marianum*, this solvent was used for the subsequent experiments. For SIL B, showing low solubility in methanol, dimethyl sulfoxide (DMSO) was employed as solvent. The stock solutions of Nano A and Nano B were prepared in DW (Figs A and B in S1 File) and stored at −20 °C until use. For human insulin fibrillation induction, aliquots of 1.5 mg/mL protein, containing 20 μM thioflavin T (ThT) and increasing concentrations of SIL A, SIL B, Nano A, or Nano B were transferred into a polystyrene 96-well plate. The plate was sealed and loaded into a microplate reader followed by incubation at 57 °C while being stirred at 250 rpm. The fluorescence intensity of ThT was recorded with excitation at 440 nm and emission at 485 nm at 20 min intervals for 15 h. For α-syn fibril formation, the protein was dissolved in phosphate buffered saline (PBS) to a final concentration of 1.5 mg/mL (∼100 μM). The aliquots of protein, containing 20 μM ThT and increasing concentrations of SIL A, SIL B, Nano A, or Nano B were incubated at 37 °C under constant stirring at 1000 rpm for 4 days. The final concentration of methanol and DMSO did not exceed 0.15% (v/v) in protein samples containing the highest concentration of compounds. The fitting of obtained data to the sigmoid curve was performed using the AmyloFit platform [47]. For Congo red (CR) binding assay, 50 μL of α-syn samples incubated without or with increasing concentrations of SIL A/Nano A for 4 days under amyloidogenic conditions was added to 950 μL of 20 μM CR and incubated for 30 min in a dark place at room temperature. Then, the absorbance spectra were recoded between 400 nm to 700 nm.

### Microscopy experiments

Fluorescence microscopy was performed to further confirm ThT fluorescence results and to investigate the morphology of formed aggregates. Briefly, 10 μL of incubated proteins was mixed with 10 μL of 50 μM ThT and incubated for 10 min in a dark place at room temperature. In some experiments, treatment of protein samples with various concentrations of bulk or nano forms of SIL A/SIL B was performed in the absence of ThT. After incubation, 10 μL of incubated samples was mixed with 10 μL of 50 μM Nile red (NR). Then, 10 μL of solutions were placed on a clean glass slide and air-dried. The images were captured on a fluorescence microscope (Zeiss, Germany) at 20 X magnification and quantified using image j software. For AFM analysis, aliquots of α-syn samples incubated in the presence of 15 and 50 μg/mL of SIL A, Nano A, SIL B, or Nano B for 4 days were removed and diluted 50-fold with DW. Then, 10 μL of diluted samples was placed on freshly cleaved mica and dried at room temperature. Images were acquired in non-contact mode using a quantitative AFM (ARA-AFM, Ara-Research Company, Iran).

### Cell viability assay

The culture and seeding of human neuroblastoma SH-SY5Y cells were performed as previously reported [36]. Cells were allowed to attach to the plate for 24 h before treatment. For cytotoxicity experiments, cells were treated with increasing concentrations (0–200 μg/mL) of SIL A/SIL B, or their respective nanoparticles. To evaluate the protective effects of compounds against cytotoxicity related to the amyloid fibrils of human insulin and α-syn, the protein samples were sterilized and incubated without or with increasing concentrations of SIL A, SIL B, Nano A, or Nano B under amyloidogenic conditions for 15 h (human insulin) and 4 days (α-syn) to produce amyloid fibrils. Then, aliquots of 20 μM protein samples were added to the cells and left for 24 h. The cells treated with glycine buffer or PBS were used as control. Cell viability was assessed using the conventional MTT reduction assay as described previously [36]. Briefly, 10 μL of MTT stock solution (5 mg/mL dissolved in PBS) was added to 100 μL of DMEM-F12 containing 10% fetal bovine serum. The solution was added to each cell followed by incubation at 37 °C for 3 h. Then, solutions were aspirated and cells were treated with DMSO for 15 min, followed by absorbance reading at 570 nm using an ELISA reader (Expert 96, Asys Hitch, Ec Austria). Results were expressed as percentage of MTT reduction relative to the control cells, assuming that the absorbance of the control cells was 100%. All measurements were made in triplicates.

### Statistical analysis

All assays were performed two or three times with triplicate repeats. The results are presented as a percentage relative to values obtained from untreated control cells and each value represents the mean ± SD (n = 3). Statistical significance was determined using an unpaired Student’s t-test, where ^#^*p* < 0.01 indicates a significant difference compared to the control group, and ^*^p < 0.01 indicates a significant difference compared to the group exposed solely to amyloid fibrils.

## Result and discussion

### Extraction of SIL A, and synthesis and characterization of Nano A and Nano B

**Fig 1:**
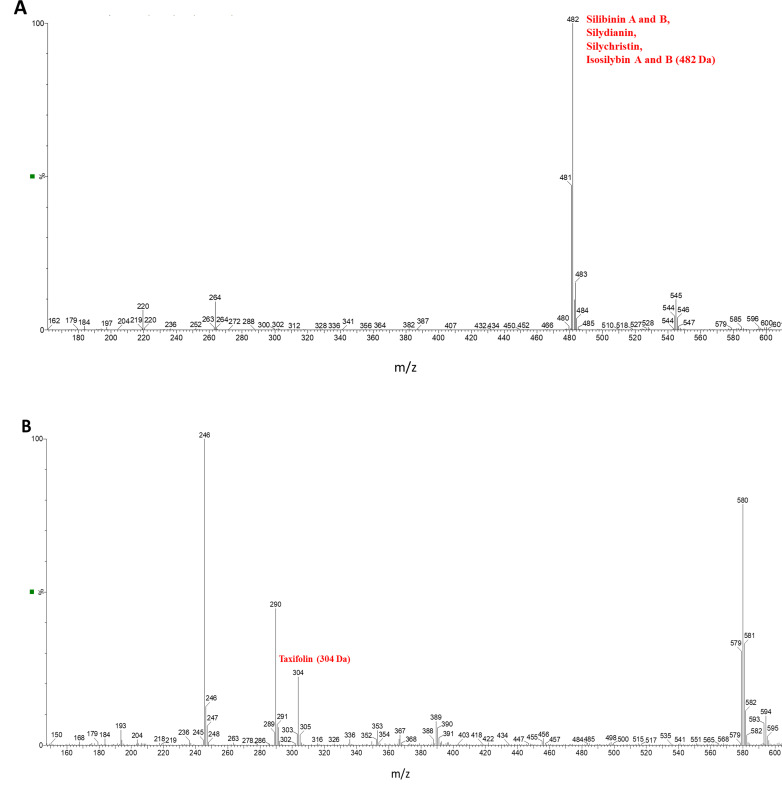
LC-MS profile of SIL A. The presence of key SIL components, including (A) silybin A and B, silydianin, silychristin, and isosilybin A and B, and (B) taxifolin are indicated.

N-hexane and methanol 80% (v/v) were used sequentially to eliminate the fatty acid/oil content (25%) of seeds and to extract SIL (SIL A), respectively [40,48]. The composition of SIL A was investigated using LC-MS, revealing the presence of key SIL components, including silybin A and B, silydianin, silychristin, and isosilybin A and B (482.0 Da) (Fig 1A and Fig C in S1 File), as well as taxifolin (304.0 Da) (Fig 1B and Fig C in S1 File).

**Fig 2:**
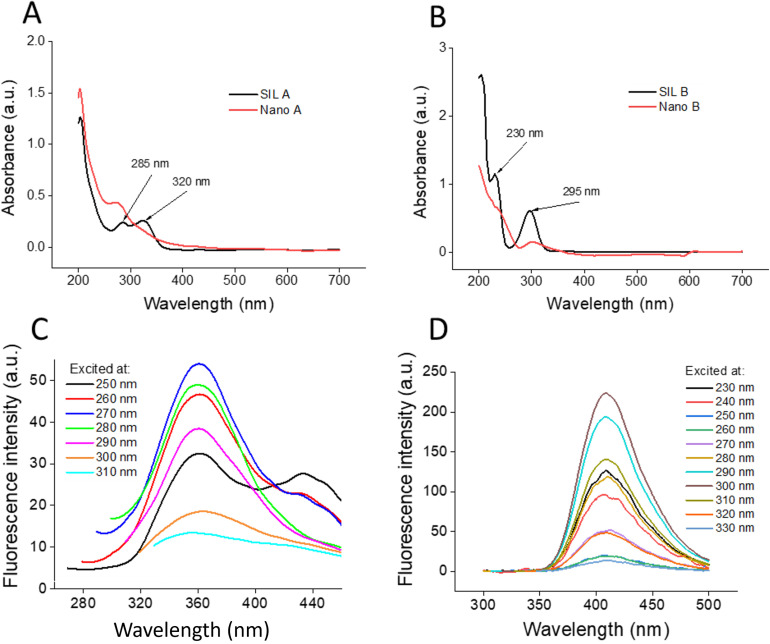
Spectroscopic characterization of SIL A, SIL B, and their respective nanoparticles. (A and B) The UV − vis spectra of SIL A/Nano A and SIL B/Nano B, respectively, prepared in a final concentration of 20 μg/mL. (C and D) The fluorescence emission spectra of SIL A and SIL B, respectively, excited at different wavelengths. Further details are provided in the text.

Due to their similar molecular weights (around 482 Da), all these compounds, except taxifolin, were observed as a single peak in Fig 1A. To further validate the presence of these constituents, HPLC analysis was conducted. The HPLC chromatogram of SIL A, depicted in Fig D in S1 File, shows several peaks corresponding to the main components of SIL. Interestingly all seven compounds, generally found in SIL extract (Fig E in S1 File), were detected in SIL A. These results are consistent with those reported in previous studies [42,49], suggesting the successful extraction of SIL. For SIL B, HPLC analysis shows 5 peaks (Fig D in S1 File), with silybin as the main component [49,50]. The attributes of each peak are provided in corresponding tables below the chromatograms. While in both SIL A and SIL B, silybin is the most component, its percentage in SIL B (70.6%) is very higher than SIL A (28.1%) (Fig D in S1 File). As depicted in Fig 2A, the UV-vis absorption spectrum of SIL A shows two peaks at around 285 nm and 320 nm, which are indicative of SIL extracted from the seeds of *Silybum marianum* [51].

**Fig 3:**
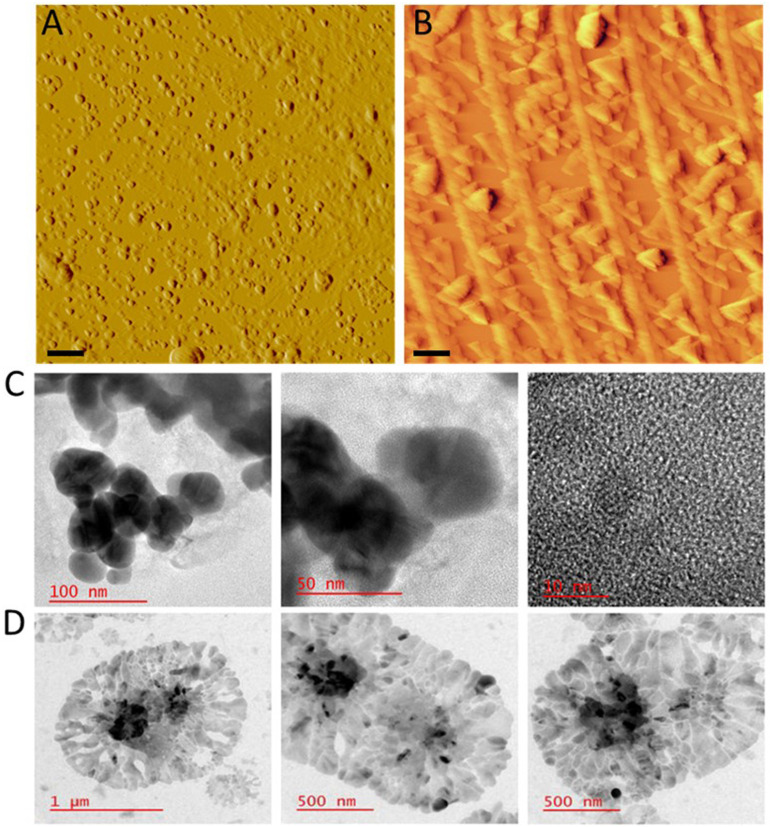
Microscopic characterization of Nano A and Nano B. (A and B) AFM images, and (C and D) HR-TEM images of Nano A and Nano B, respectively. In AFM images the scale bars represent 500 nm.

For SIL B, the UV-vis absorption spectrum showed a prominent peak centered at around 295 nm and a peak at 230 nm (Fig 2B), which is in agreement with the absorption spectra of silybin [52,53] as the main component of SIL [50], and in accord with HPLC results (Fig D in S1 File). Upon nanonization, however, a significant change in the absorption spectra of both SILs was observed, characterized by a considerable enhancement in the absorbance intensity with a blue shift for SIL A (Fig 2A), and a notable decrease in the absorption spectrum of SIL B (Fig 2B). A significant characteristic of SIL A and SIL B is their excitation wavelength-dependent fluorescence. As depicted in Fig 2C, excitation of SIL A at different wavelengths resulted in a range of emission spectra reaching a maximum at 360 nm when excited at 270 nm. The maximum fluorescence emission of SIL B was about 410 nm when excited at 300 nm (Fig 2D). Upon nanonization, however, the pattern and the intensity of fluorescence signal was significantly changed. As shown in Fig F in S1 File, the maximum fluorescence of Nano A was about 440 nm when excited at 250 nm. For SIL B, nanonization caused a significant diminish of fluorescence intensity so that we couldn’t see any detectable emission peak when excitation was performed at different wavelengths (Fig F in S1 File). It is believed that ordered sp^2^ domains isolated within sp^3^ C − O and sp^2^ C − O structures are responsible for the absorption and fluorescence properties of polyphenols [37], and any change in this pattern can alter spectroscopic properties of these compounds. Therefore, we suggest that fusion of polyphenolic structures during nanonization can change the number of these ordered domains leading to such absorption/fluorescence alterations. Finally, AFM and HR-TEM experiments were performed to confirm the formation of nanoparticles and to examine their morphological features. As shown in Fig 3A, Nano A exhibits a spherical morphology with an average diameter of less than 100 nm.

The homogenous, round structure of SIL A nanoparticles was further confirmed by HR-TEM images (Fig 3C). In the case of SIL B, AFM and HR-TEM images revealed a heterogenous and sheet morphology with different diameters ranging from 200 nm to 1500 nm (Figs 3B and D). From the images it may conclude that both lateral and vertical interactions are involved in the formation of SIL B nanoparticles (Figs 3B and D). While we used a similar protocol for SIL A and SIL B nanoparticle preparation (see the materials and methods section), the morphology of nanoparticles was completely different (Fig 3). A possible explanation for this observation may be related to the composition and chemical components of these compounds. SIL A, extracted from the *Silybum marianum* seeds, is a heterogeneous solution containing various organic and inorganic components. We believe that the presence of these components may interfere with the interaction of main components of SIL A, leading to the formation of nanoparticles with defined size and morphology. Based on the HPLC results (Fig D in S1 File), SIL B is a relatively homogenous solution containing silybin as the main component. Moreover, the addition of CA as a cross-linking agent to increase the efficiency of nanoparticle production may result in formation of large nanoparticles with a sheet morphology. Despite the presence of some impurities in SIL extracted from the seeds of *Silybum marianum*, the results obtained by UV-vis spectroscopy, LC-MS, HPLC, AFM, and HR-TEM confirm that the extraction of main components of natural SIL and the synthesis of SIL A and SIL B nanoparticles are successfully performed.

### Antioxidant activity of SIL A, SIL B, and their respective nanoparticles

**Fig 4:**
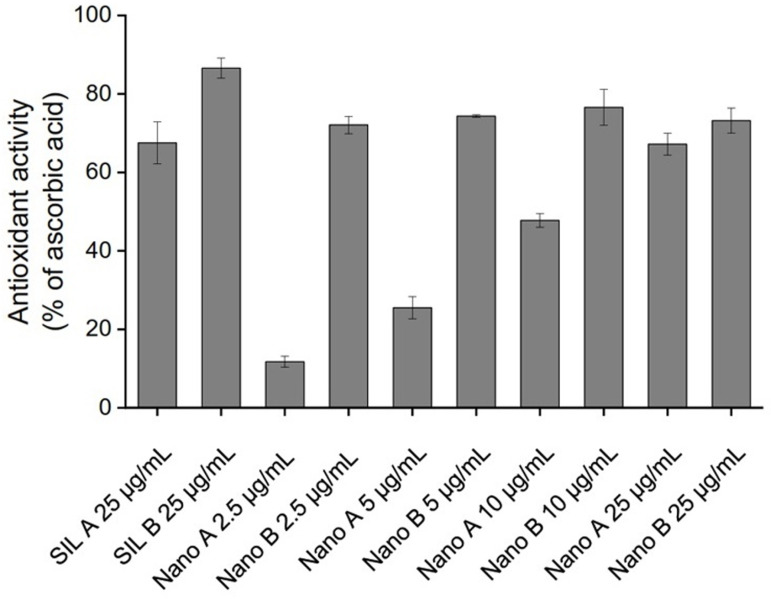
DPPH-based antioxidant activity of SIL A, SIL B, and their respective nanoparticles. The results are calculated as a fraction of 25 µg/mL ascorbic acid.

It has been shown that many therapeutic effects of natural polyphenols, including their neuroprotective features, are related to their ability to scavenge free radical species [54]. Thus, a DPPH-based antioxidant assay was employed to compare the antioxidant effects of SIL A with SIL B and to investigate the impact of nanonization on the free radical scavenging properties of these compounds. While both SIL A and SIL B showed the capacity to scavenge the DPPH^•^ radicals, SIL B was more effective (Fig 4), presumably due to its higher silybin content (Fig D in S1 File), which accounts for the antioxidant properties of SIL [20,50].

As shown in Fig 4, nanonization had little, if any, impact on the free radical scavenging effects of SIL B. A possible explanation for this observation may be related to the decreased hydroxyl functional groups during nanoparticle production, which can reduce the capacity of SIL B to scavenge free radical species. While both bulk and nano forms of SIL A and SIL B exhibit significant antioxidant activity (Fig. 4) without any significant cytotoxicity (Fig G in S1 File), but due to their higher surface/volume ratio, we believe that Nano A/B, in comparison to their respective bulk forms, have higher capacity to interact and bind to amyloidogenic species leading to more effective inhibition of the assembly process of proteins [36–39,55,56]. Accordingly, we considered these nanoparticles as suitable candidates for the subsequent anti-amyloidogenic experiments.

### Effect of SIL A, SIL B, and their respective nano forms on amyloid fibrillation of human insulin

**Fig 5:**
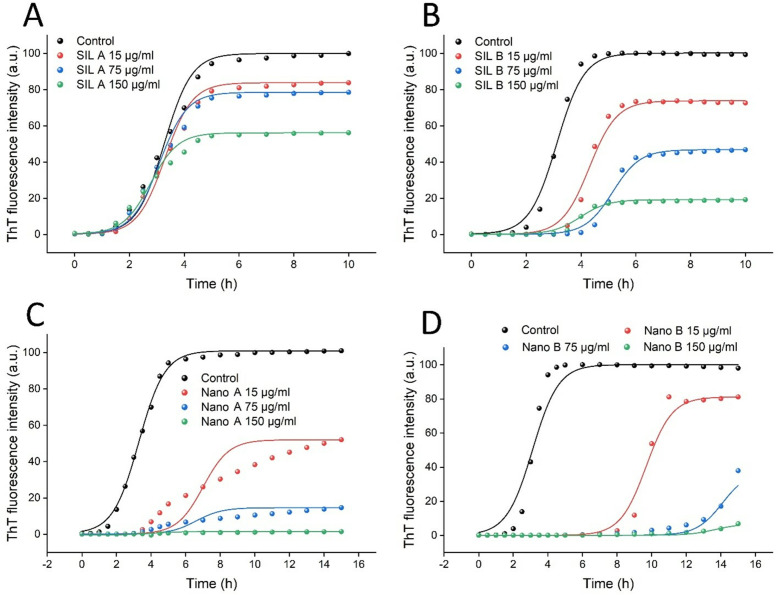
The effect of (A) SIL A, (B) SIL B, (C) Nano A, and (D) Nano B on the kinetics of human insulin amyloid fibrillation monitored by increasing fluorescence intensity of ThT. Protein samples were incubated at 57 °C while begin stirred at 250 rpm. The ThT fluorescence signal was recorded in 20 min intervals, but for simplifying the figure, signals in the plateau phase are indicated at 1 h intervals. The solid lines show fitting developed by AmyloFit [47].

Kinetics of human insulin fibrillation in the absence and presence of increasing concentrations of SIL A or SIL B (15, 75, and 150 µg/mL) are shown in Figs 5A and B, respectively, indicating a concentration-dependent decrease in ThT fluorescence intensity.

**Fig 6:**
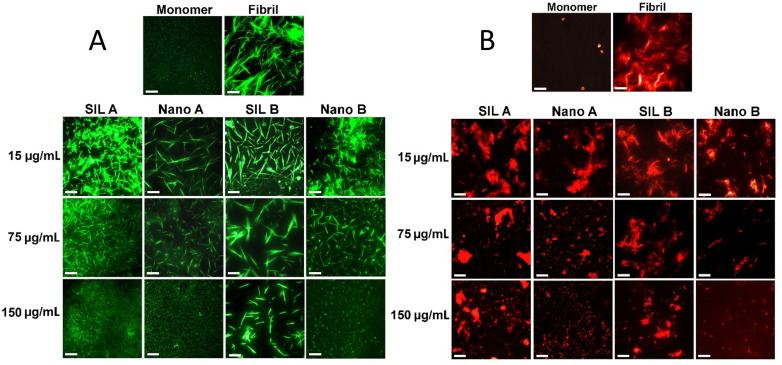
The effect of SIL A/Nano A and SIL B/Nano B on (A) amyloid fibrillation and (B) surface hydrophobicity of human insulin monitored by ThT and NR fluorescence microscopies, respectively. The protein samples were incubated under amyloidogenic condition either alone or with increasing concentrations of compounds for 15 h, followed by microscopy imaging. The scale bars represent 500 nm.

However, the efficacy of SIL B was remarkably higher than SIL A. Moreover, the nucleation phase was prolonged only in protein samples incubated with SIL B (Fig 5B and Table A in S1 File). While SIL contains a mixture of polyphenolic flavonolignans (Fig E in S1 File), silybin is the most active gradient and responsible for the most biological, especially antioxidant and anti-amyloidogenic, activities of SIL [20,29,31,33,50,57]. Thus, we suggest that the higher anti-amyloidogenic activity of SIL B may be attributed to its higher silybin content, as indicated by HPLC data (Fig D in S1 File). Similar to our results, previous reports indicate prolongation of the nucleation phase of various peptides and proteins induced by silybin [29,31,34]. Upon nanoparticle formation, the capacity of both Nano A and Nano B to prevent amyloid fibril formation of human insulin was significantly enhanced in a concentration-dependent manner. As shown in Figs 5C and D, in samples containing the highest concentration of either Nano A or Nano B, no detectible ThT fluorescence signal was observed, even when incubation under amyloidogenic condition was continued up to 15 h. At low to moderate concentrations, however, the capacity of Nano B to attenuate the formation of primary nuclei was markedly higher than Nano A (Figs 5C and D). As shown in Table A in S1 File, the lag time was extended from 2.02 ± 0.04 in control sample to 8.08 ± 0.54 and 12.37 ± 0.31 in samples incubated with 15 and 75 µg/mL Nano B, respectively. These results were further supported by ThT fluorescence microscopy. As shown in Fig 6A and Fig H in S1 File, the amount and size of amyloid fibrils decreased dose-dependently by increasing the concentration of compounds.

This decrease was more significant in samples containing SIL nanoparticles, especially Nano B. As shown in Fig 6A, the formation of human insulin amyloid fibrils was inhibited entirely in the presence of 150 µg/mL Nano B, and instead, very small structures were produced. We believe that the higher capacity of Nano B in attenuating the assembly process of human insulin may be related to their morphological features. On the other hand, thanks to their sheet morphology (Fig 3D) and high surface/volume ratio, Nano B can interact more effectively with monomers of human insulin and thereby trap a greater number of amyloidogenic species on their surface. This would decrease the bulk concentration of protein, and consequently, protein-protein interactions required for the growth of amyloidogenic species leading to more effective inhibition of amyloid fibril formation. On the contrary, Nano A with a smaller size (Fig 3C) and a low surface/volume ratio, may not be able to bind and hold monomeric species as effective as Nano B to inhibit the conformational changes required for the primary nuclei formation (Fig 5C and Table A in S1 File). On the contrary, by interfering with the elongation phase of amyloid fibrillation, Nano A may prevent the growth of amyloid fibrils leading to the formation of short fibrils (Fig 6A). This size-dependent modulating effect of nanoparticles has been reported by previous studies [55,56], suggesting that the surface area of nanoparticles can influence the aggregation pathway and their mechanism of action. Since exposure of hydrophobic regions as a result of misfolding and aggregation of amyloidogenic peptides/proteins is a crucial and common step in the course of amyloid fibrillation and cytotoxicity [58], changes on the surface hydrophobicity of protein samples incubated alone or in the presence of increasing concentrations of SIL A or SIL B, or their respective nano forms, were measured using NR fluorescence microscopy. As shown in Fig 6B, incubation of human insulin under amyloidogenic condition resulted in a significant enhancement in NR fluorescence, indicating the formation of amyloid fibrils. In accordance with ThT results, the presence of SIL A or SIL B, or their respective nanoparticles, remarkably decreased the fluorescence intensity of NR in a concentration-dependent manner (Fig 6B and Fig H in S1 File). However, the efficacy of nanoparticles, particularly Nano B, in preventing the exposure of hydrophobic surfaces was higher than their bulk forms, which is in accord with ThT fluorescence and microscopy data (Figs 5 and 6A).

### Effect of SIL A, SIL B, and their respective nano forms on amyloid fibrillation of α-synuclein

Previous reports indicate that the capacity of polyphenols to modulate amyloid fibrillation and their mechanism of action, may be specific and dependent on the amino acid sequence of target polypeptide [38,59]. Human insulin is a structured peptide (containing 51 amino acids) composed of more than 90% α-helices [60]. To investigate the potency of SILs and their respective nanoparticles in modulating the fibrillation of natively unfolded peptides/proteins, which are mainly involved in NDs, the effect of these compounds in inhibiting the assembly process of α-syn was examined. The kinetics of α-syn fibrillation alone and in the presence of various concentrations (5, 25, and 50 µg/mL) of SIL A, SIL B, or their respective nanoforms was monitored by ThT fluorescence measurement.

**Fig 7:**
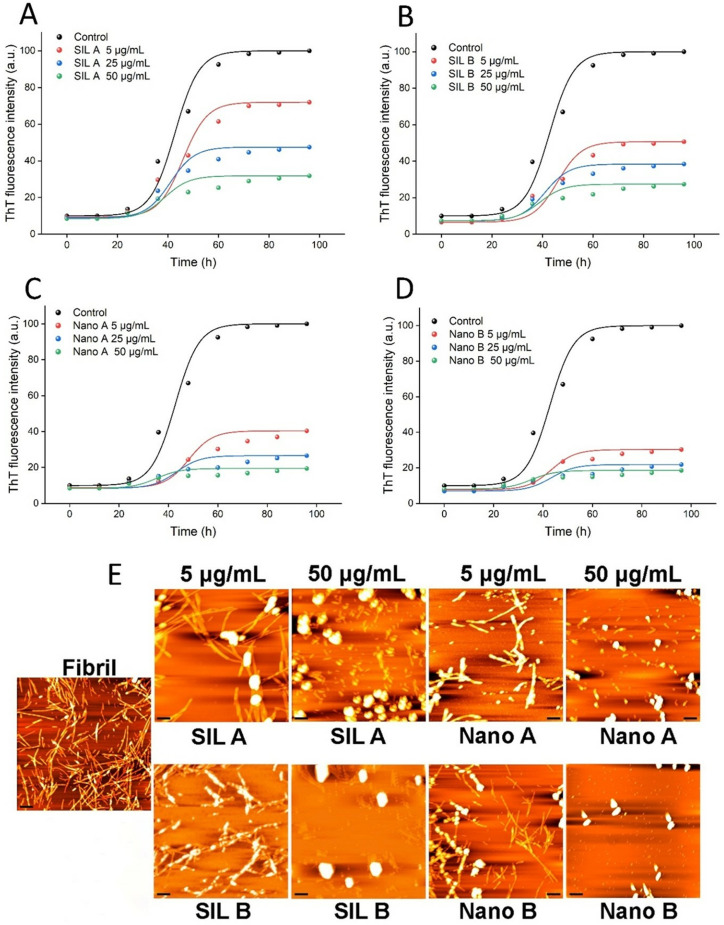
The effect of SIL A, SIL B, Nano A, and Nano B on the amyloid fibrillation of **α-syn.** (A-D) The kinetics of α-syn amyloid fibril formation monitored by measuring fluorescence intensity of ThT in the presence of increasing concentrations of SIL A, SIL B, Nano A, and Nano B, respectively. The solid lines show fitting developed by AmyloFit [47]. (E) AFM images of α-syn samples incubated alone or with increasing concentrations of SIL A, SIL B, Nano A, and Nano B for 96 h. The scale bars represent 500 nm.

Similar to the results obtained for human insulin (Fig 5), the potency of SIL B to inhibit α-syn fibrillogenesis was higher than SIL A (Figs 7A and B), and nanonization enhanced the inhibitory effects of both SILs (Figs 7C and D). But, in contrast to human insulin, inhibition of α-syn amyloid fibril formation by both SILs or their respective nanoforms was promoted with a slight change in the nucleation phase of fibrillation (Figs 7A-D and Table B in S1 File). As a complementary evaluation of amyloid fibril formation, CR absorbance measurement was employed to further monitor the presence of β-sheet structures associated with amyloid fibrils. As shown in Fig I in S1 File, a marked enhancement in CR absorbance along with a significant red shift was observed for α-syn incubated alone, indicating the presence of large amount of amyloid fibrils. The presence of SIL A or SIL B, and in particular their respective nanoforms, considerably inhibited this enhancement (Fig I in S1 File), indicating the improved inhibitory effects of both SILs upon nanonization. This data was further confirmed by AFM and ThT fluorescence microscopy. As shown in Fig 7E and Fig J in S1 File, in the absence of tested compounds, well-defined mature fibrils were formed. The presence of SIL A or SIL B led to a dose-dependent decrease in the amount of amyloid fibrils along with the formation of amorphous aggregates in samples containing 50 μg/mL of compounds. The extent of inhibition was even higher in samples treated with nanoforms of SIL A or SIL B. As depicted in Fig 7E and Fig J in S1 File, no fibrils were observed in the presence of 50 μg/mL nano SILs and instead, some small amorphous aggregates were detected. These results demonstrate that the fibrillation-modulating activity of SIL A, SIL B, Nano A, and Nano B is not restrict to structured proteins and can be applied to natively unfolded proteins involving in amyloid-related human diseases. According to the obtained results, we may conclude that the mechanism by which SIL A and SIL B, or their respective nanoforms modulate the amyloid fibrillation of human insulin and α-syn is different. For instance, treatment of human insulin with increasing concentrations of SIL A/SIL B, and particularly Nano A/Nano B, prevented formation of mature amyloid fibrils with the appearance of short fibrils (Fig 6A). In the case of α-syn, we did not observe any fibrillar structure, but some amorphous aggregates were appeared. In the presence of 50 µg/mL Nano A/Nano B, even these amorphous aggregates disappeared, and instead, some small globular amorph structures were observed (Fig 7E and Fig J in S1 File). This conclusion is in accord with previous reports indicating that the mechanism by which polyphenolic compounds inhibit fibrillogenesis of proteins is dependent on the amino acid sequence and the conformation of target polypeptide [38,59]. In accord with this conclusion, it has been shown that the molecular mechanisms underpinning the fibrillogenesis of structured and natively unfolded proteins are different [61], which may lead to sequence-specific mechanisms for inhibition of aggregation.

### Effect of SIL A, SIL B, and their respective nano forms on the cytotoxicity induced by human insulin and α-syn amyloid fibrils

**Fig 8:**
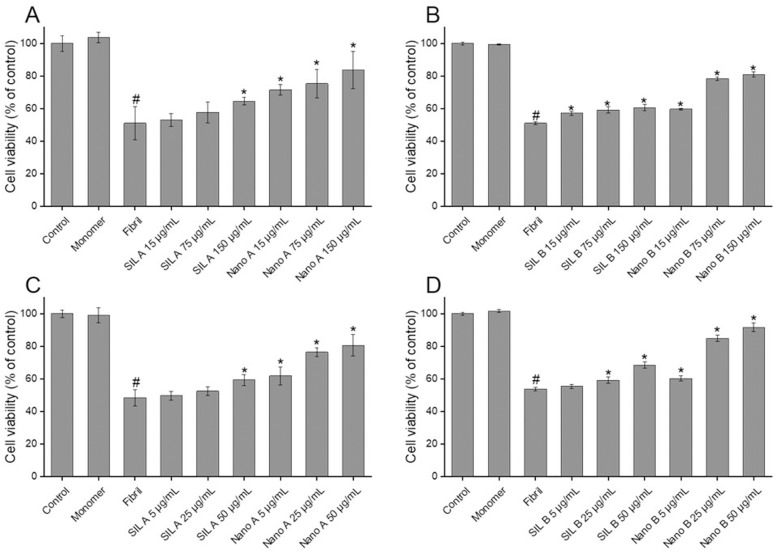
Protective effects of SIL A, SIL B, and their respective nanoparticles against cytotoxicity induced by human insulin and **α-syn aggregates measured by MTT assay.** (A and B) Cytotoxicity evaluation of human insulin and (C and D) α-syn aggregates produced in the absence or presence of increasing concentrations of SIL A, SIL B, or their respective nanoparticles, respectively. ^#^p < 0.01, significantly different from control cells. ^*^p < 0.01, significantly different from cells exposed only to human insulin or α-syn amyloid fibrils.

To investigate the capacity of tested compounds in attenuating cytotoxicity associated with amyloid fibrils, the human neuroblastoma SH-SY5Y cells were exposed to the amyloidogenic species aged in the absence or presence of increasing concentrations of SIL A, SIL B, or their respective nanoparticles for 24 h and the viability of cells was measured by MTT assay. As shown in Fig G in S1 File, we didn’t observe any cytotoxicity corresponding to SIL A/SIL B, or their respective nanoparticles when they applied at concentrations up to 200 μg/mL. According to our previous report [38], 20 µM amyloid fibrils was selected for the cytotoxicity experiments. While the monomeric forms of human insulin and α-syn were totally non-toxic, treatment of SH-SY5Y cells with 20 µM amyloid fibrils for 24 h resulted in a considerable decrease in the viability of cells (Fig 8).

The presence of either SIL A or SIL B, or their respective nanoparticles, decreased significantly the cytotoxicity of protein aggregates in a dose-dependent manner (Fig 8). However, the capacity of Nano A and Nano B in preventing cytotoxicity was higher than their respective bulk forms, with more than 80% viability in cells treated with protein samples containing the highest concentration of Nano A or Nano B (Fig 8). Based on these results we may suggest that despite difference in the mechanism of action of nanoparticles, both Nano A and Nano B are able to attenuate cytotoxicity associated with human insulin and α-syn fibrils in a concentration-dependent manner.

## Conclusions

Almost all reports on the neuroprotective effects of SIL [21–28] and anti-amyloidogenic properties of silybin [29–31,33,34], have used the commercial forms of these compounds. In this study, for the first time, the potential of natural SIL (SIL A) and its nano form as amyloid fibrillation inhibitors was investigated using human insulin and α-syn proteins. The obtained results demonstrated that SIL A has a certain inhibitory effect on both proteins, and that nanonization can increase the anti-amyloidogenic effects of SIL A. These results are in accord with our previous reports and in accord with the surface assistance model [36,37,55,56]. Moreover, we found that the mechanism by which these compounds modulate the amyloid fibrillation of proteins may be different and dependent on the amino acid sequence and conformational properties of target peptide/protein. Considering to any significant cytotoxicity (Fig G in S1 File) and potential of Nano A to bind and detect protein aggregates (Fig K in S1 File), along with their fluorescence properties (Fig 2C and D), we may introduce these nanoparticles as new potential candidate for detection of intracellular protein aggregates. However, further *in vitro* and *in vivo* experiments are needed to validate these findings.

## Supporting information

S1 File **Table A**. Effect of increasing concentrations of SIL A/Nano A, and SIL B/Nano B on the kinetic parameters of human insulin fibrillation determined by ThT fluorescence assay. Table B. Effect of increasing concentrations of SIL A/Nano A, and SIL B/Nano B on the kinetic parameters of α-syn fibrillation determined by ThT fluorescence assay. Fig A. The obtained powders of (**A**) SIL A and (**B**) Nano A. (**C**) Solutions (2 mg/mL) of SIL A and Nano A dissolved in methanol and DW, respectively. Fig B. The obtained powders of (**A**) SIL B and (**B**) Nano B. (**C**) Solutions (2 mg/mL) of SIL B and Nano B dissolved in DMSO and DW, respectively. Fig C. Ion spectra of SIL A with a molecular mass of (**A**) 482 corresponding to silybin A and B, silydianin, silychristin, isosilybin A and B, and (**B**) 304 corresponding to taxifolin. Insets indicate isotope distribution of components. Fig D. HPLC chromatogram of (**A**) SIL A and (**B**) SIL B. The tables show attributes of HPLC chromatograms, including the retention time and concentration of each component. While in both SIL A and SIL B, silybin is the most constituent, but its amount in SIL B (70.6%) is very higher than SIL A (28.1%). Fig E. Chemical structure of main constituents of SIL extract. Fig F. Fluorescence emission spectra of (**A**) Nano A and (**B**) Nano B, excited at different wavelengths. Fig G. Cytotoxicity of SIL A, SIL B, and their respective nanoparticles evaluated by MTT-based viability assay. Fig H. Quantification of (**A**) ThT and (**B**) NR fluorescence intensities of human insulin samples incubated in the presence of various concentrations of SIL A, SIL B, Nano A, and Nano B. The concentrations of compounds are μg/mL. The results are the average of 5 fluorescence images. Fig I. Congo red (CR) binding absorption spectra of α-syn samples incubated alone or with increasing concentrations of SIL A, SIL B, Nano A, and Nano B. CR absorbance in the presence of PBS and α-syn monomer are also indicated. Fig J. ThT fluorescence microscopy images of α-syn samples incubated alone or with increasing concentrations of SIL A, SIL B, Nano A, and Nano B for 96 h. Graph shows quantification of ThT fluorescence images. The concentrations of compounds are μg/mL. The results are the average of 5 fluorescence images. The scale bars represent 500 nm. Fig K. Co-staining of human insulin and α-syn amyloid aggregates with ThT and Nano A. The left and middle panels indicate green and red fluorescence of amyloid fibrils stained by ThT and Nano A, respectively. The right panels are the merged images of ThT and Nano A indicating co-localization of ThT and Nano A on amyloid fibrils. The scale bar represents 500 nm. The co-staining experiments were done according to our previous report [36].
(DOCX)
