# Supplementary material for: Inhibition of cytotoxic fibril formation of α-synuclein and human insulin by Silymarin from the Silybum marianum
Source: PLoS One. 2025 May 2;20(5):e0320283. doi: 10.1371/journal.pone.0320283 (PMC12047757; doi:10.1371/journal.pone.0320283)
Supplement: S1 File — Table A. Effect of increasing concentrations of SIL A/Nano A, and SIL B/Nano B on the kinetic parameters of human insulin fibrillation determined by ThT fluorescence assay. Table B. Effect of increasing concentrations of SIL A/Nano A, and SIL B/Nano B on the kinetic parameters of α-syn fibrillation determined by ThT fluorescence assay. Fig A. The obtained powders of (A) SIL A and (B) Nano A. (C) Solutions (2 mg/mL) of SIL A and Nano A dissolved in methanol and DW, respectively. Fig B. The obtained powders of (A) SIL B and (B) Nano B. (C) Solutions (2 mg/mL) of SIL B and Nano B dissolved in DMSO and DW, respectively. Fig C. Ion spectra of SIL A with a molecular mass of (A) 482 corresponding to silybin A and B, silydianin, silychristin, isosilybin A and B, and (B) 304 corresponding to taxifolin. Insets indicate isotope distribution of components. Fig D. HPLC chromatogram of (A) SIL A and (B) SIL B. The tables show attributes of HPLC chromatograms, including the retention time and concentration of each component. While in both SIL A and SIL B, silybin is the most constituent, but its amount in SIL B (70.6%) is very higher than SIL A (28.1%). Fig E. Chemical structure of main constituents of SIL extract. Fig F. Fluorescence emission spectra of (A) Nano A and (B) Nano B, excited at different wavelengths. Fig G. Cytotoxicity of SIL A, SIL B, and their respective nanoparticles evaluated by MTT-based viability assay. Fig H. Quantification of (A) ThT and (B) NR fluorescence intensities of human insulin samples incubated in the presence of various concentrations of SIL A, SIL B, Nano A, and Nano B. The concentrations of compounds are μg/mL. The results are the average of 5 fluorescence images. Fig I. Congo red (CR) binding absorption spectra of α-syn samples incubated alone or with increasing concentrations of SIL A, SIL B, Nano A, and Nano B. CR absorbance in the presence of PBS and α-syn monomer are also indicated. Fig J. ThT fluorescence microscopy images of α-syn samples [file pone.0320283.s001.docx]

**Supporting information**

**Inhibition of cytotoxic fibril formation of α-synuclein and human insulin by silymarin from the *silybum marianum***

Beitollah Moosakhani^1#^, Mahshid Taleb^1#^, Zahra Mahmoudi Eshkaftaki^1^, Nasser Nikfarjam^2^, Azam Serajian^2^, [Mohammad Bagher Shahsavani](https://www.nature.com/articles/s41598-024-54464-4#auth-Mohammad_Bagher-Shahsavani-Aff4)^3^, Ali Akbar Meratan^1*^

^1^ Department of Biological Sciences, Institute for Advanced Studies in Basic Sciences (IASBS), Zanjan 45137-66731, Iran.

^2^ Department of Chemistry, Institute for Advanced Studies in Basic Sciences (IASBS), Zanjan 45137-66731, Iran.

^3^ Protein Chemistry Laboratory (PCL), Department of Biology, College of Sciences, Shiraz University, Shiraz 7196484334, Iran.

^*^Corresponding Author:

Ali Akbar Meratan

[a.meratan@iasbs.ac.ir](mailto:a.meratan@iasbs.ac.ir); [a.meratan@gmail.com](mailto:a.meratan@gmail.com)

^#^ These authors contributed equally

**S1 File Table A.** Effect of increasing concentrations of SIL A/Nano A, and SIL B/Nano B on the kinetic parameters of human insulin fibrillation determined by ThT fluorescence assay.

|  | Lag time (h) | t_50_ (h) | Amplitude |
| --- | --- | --- | --- |
| Control | 2.02 ± 0.04 | 3.41 ± 0.04 | 100 ± 2.77 |
| SIL A 15 µg/mL | 2.03 ± 0.11 | 3.25 ± 0.45 | 83.85 ± 1.56 |
| SIL A 75 µg/mL | 2.02 ± 0.02 | 3.02 ± 0.16 | 78.55 ± 2.04 |
| SIL A 150 µg/mL | 2.00 ± 0.06 | 2.71 ± 0.55 | 56.19 ± 1.97 |
| Nano A 15 µg/mL | 4.86 ± 0.23 | 6.87 ± 0.82 | 52.01 ± 2.11 |
| Nano A 75 µg/mL | 5.38 ± 0.66 | 6.11 ± 0.71 | 28.41 ± 1.36 |
| Nano A 150 µg/mL | -^a^ | -^a^ | 1.46 ± 0.04 |
| SIL B 15 µg/mL | 3.32 ± 0.09 | 4.26 ± 0.11 | 72.69 ± 0.74 |
| SIL B 75 µg/mL | 4.41 ± 0.72 | 5.63 ± 0.11 | 46.85 ± 1.28 |
| SIL B 150 µg/mL | 3.49 ± 0.13 | 4.06 ± 0.09 | 19.22 ± 1.59 |
| Nano B 15 µg/mL | 8.08 ± 0.54 | 9.87 ± 0.12 | 81.18 ± 1.93 |
| Nano B 75 µg/mL | 12.37 ± 0.31 | 14.11 ± 0.13 | 53.19 ± 1.52 |
| Nano B 150 µg/mL | -^a^ | 13.54 ± 0.28 | 10.03 ± 0.14 |

^a^ Due to significant inhibition of aggregation, the lag time and t_50_ could not be determined.

**S1 File Table B.** Effect of increasing concentrations of SIL A/Nano A, and SIL B/Nano B on the kinetic parameters of α-syn fibrillation determined by ThT fluorescence assay.

|  | Lag time (h) | t_50_ (h) | Amplitude |
| --- | --- | --- | --- |
| Control | 26.64 ± 39 | 40.2 ± 2.77 | 100 ± 1.72 |
| SIL A 15 µg/mL | 29.28 ± 1.05 | 41.4 ± 2.11 | 71.9606 ± 1.04 |
| SIL A 75 µg/mL | 27.69 ± 1.22 | 36.41 ± 2.73 | 47.48295 ± 2.06 |
| SIL A 150 µg/mL | 27.21 ± 2.73 | 32.61 ± 1.58 | 31.81918 ± 1.99 |
| Nano A 15 µg/mL | 32.84 ± 0.79 | 32.57 ± 2.66 | 34.40669 ± 2.33 |
| Nano A 75 µg/mL | 33.61 ± 1.27 | 30.17 ± 1.38 | 26.59938 ± 1.41 |
| Nano A 150 µg/mL | 28.53 ± 1.27 | 24.21 ± 0.78 | 19.46582 ± 0.94 |
| SIL B 15 µg/mL | 33.71 ± 1.96 | 43.24 ±2.43 | 50.68185 ± 3.42 |
| SIL B 75 µg/mL | 32.17 ± 1.62 | 37.47 ± 1.87 | 38.41846 ± 2.56 |
| SIL B 150 µg/mL | 33.55 ± 2.71 | 31.71 ± 1.44 | 27.41541 ± 2.87 |
| Nano B 15 µg/mL | 33.73 ± 1.44 | 32.28 ± 0.56 | 30.29165 ± 1.99 |
| Nano B 75 µg/mL | 36.92 ± 1.51 | 39.94 ± 0.87 | 21.83809 ± 2.56 |
| Nano B 150 µg/mL | 31.82 ± 1.73 | 14.31 ± 0.44 | 13.64573 ± 2.11 |

**
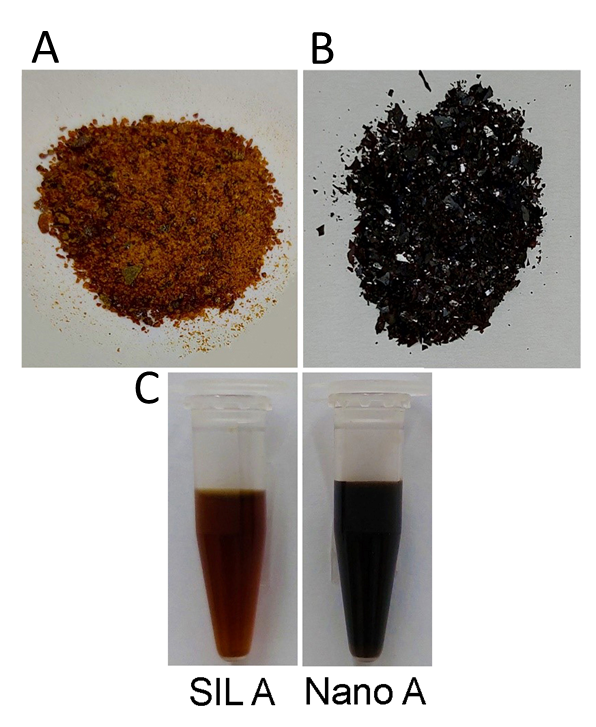
**

**S1 File Fig A.** The obtained powders of (A) SIL A and (B) Nano A. (C) Solutions (2 mg/mL) of SIL A and Nano A dissolved in methanol and DW, respectively.


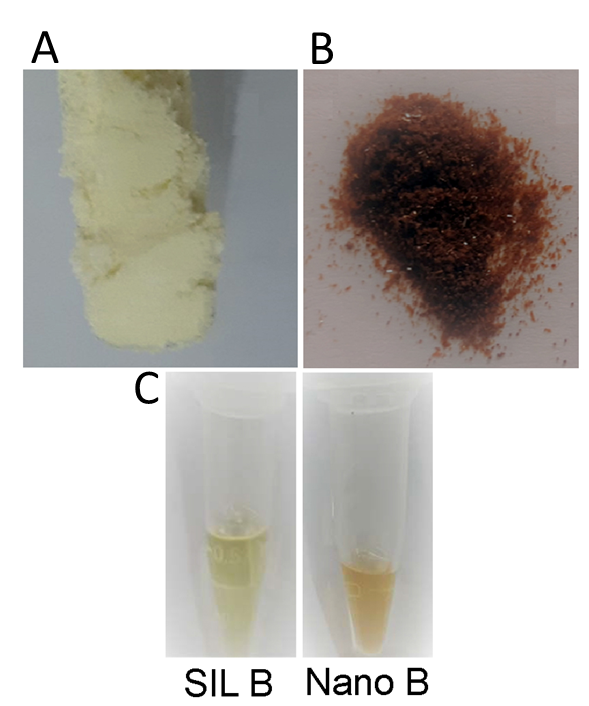


**S1 File Fig B.** The obtained powders of (A) SIL B and (B) Nano B. (C) Solutions (2 mg/mL) of SIL B and Nano B dissolved in DMSO and DW, respectively.

**
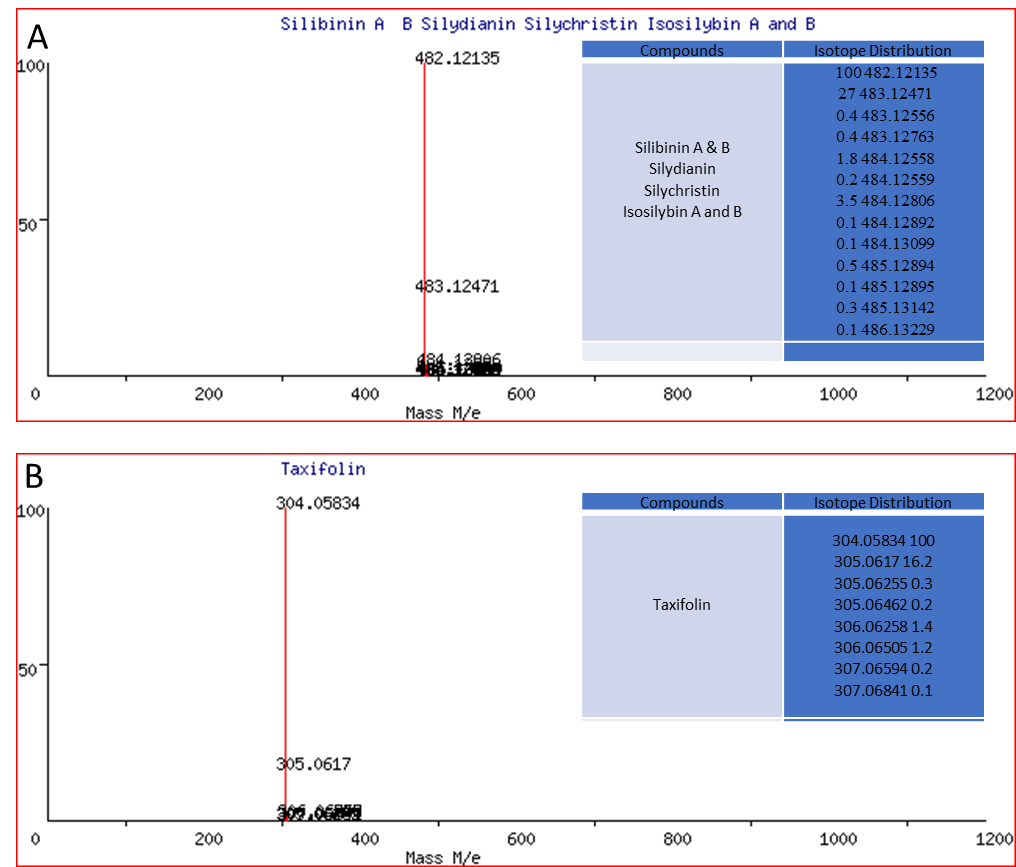
**

**S1 File Fig C.** Ion spectra of SIL A with a molecular mass of (A) 482 corresponding to silybin A and B, silydianin, silychristin, isosilybin A and B, and (B) 304 corresponding to taxifolin. Insets indicate isotope distribution of components.


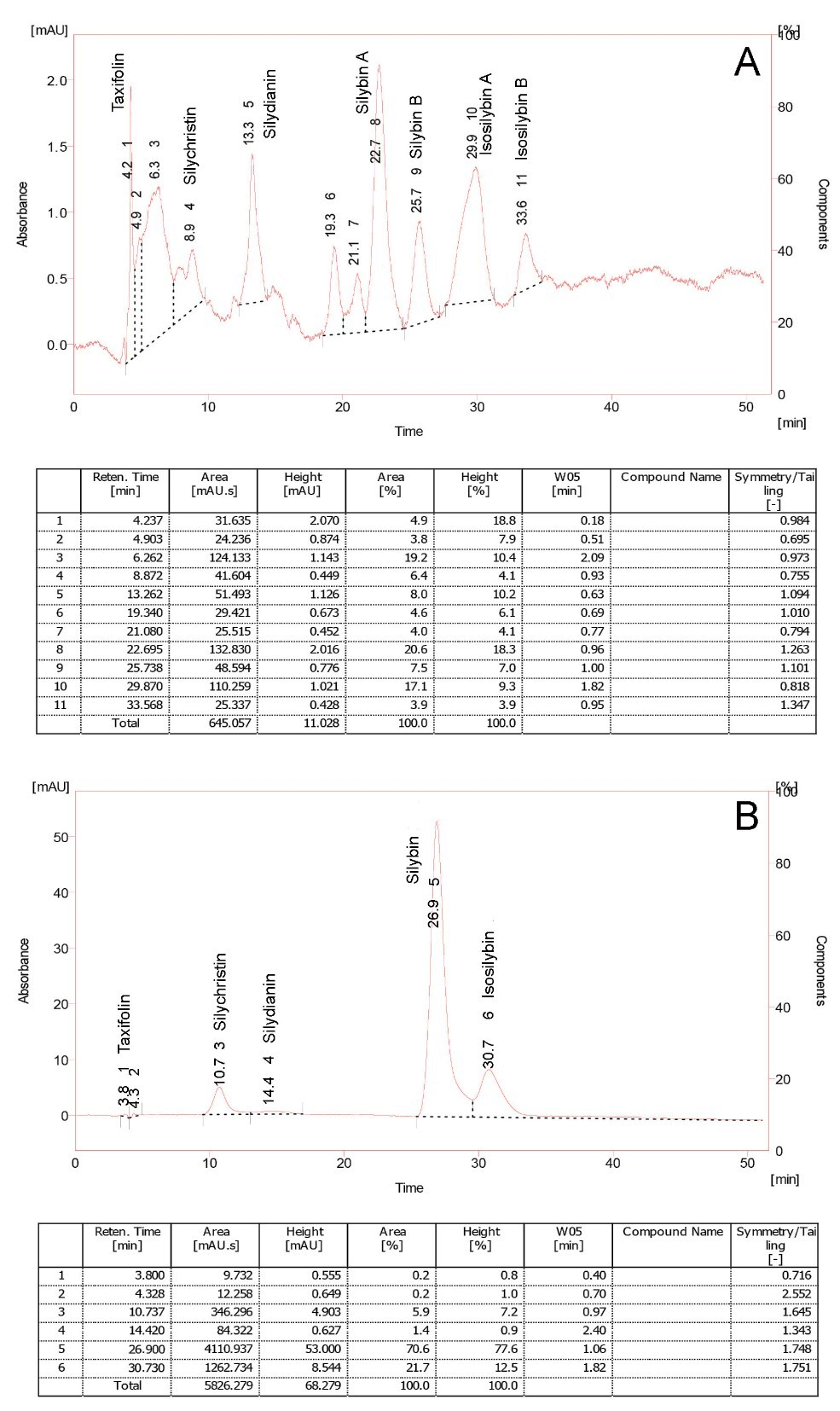


**S1 File** **Fig D.** HPLC chromatogram of (A) SIL A and (B) SIL B. The tables show attributes of HPLC chromatograms, including the retention time and concentration of each component. While in both SIL A and SIL B, silybin is the most constituent, but its amount in SIL B (70.6%) is very higher than SIL A (28.1%).


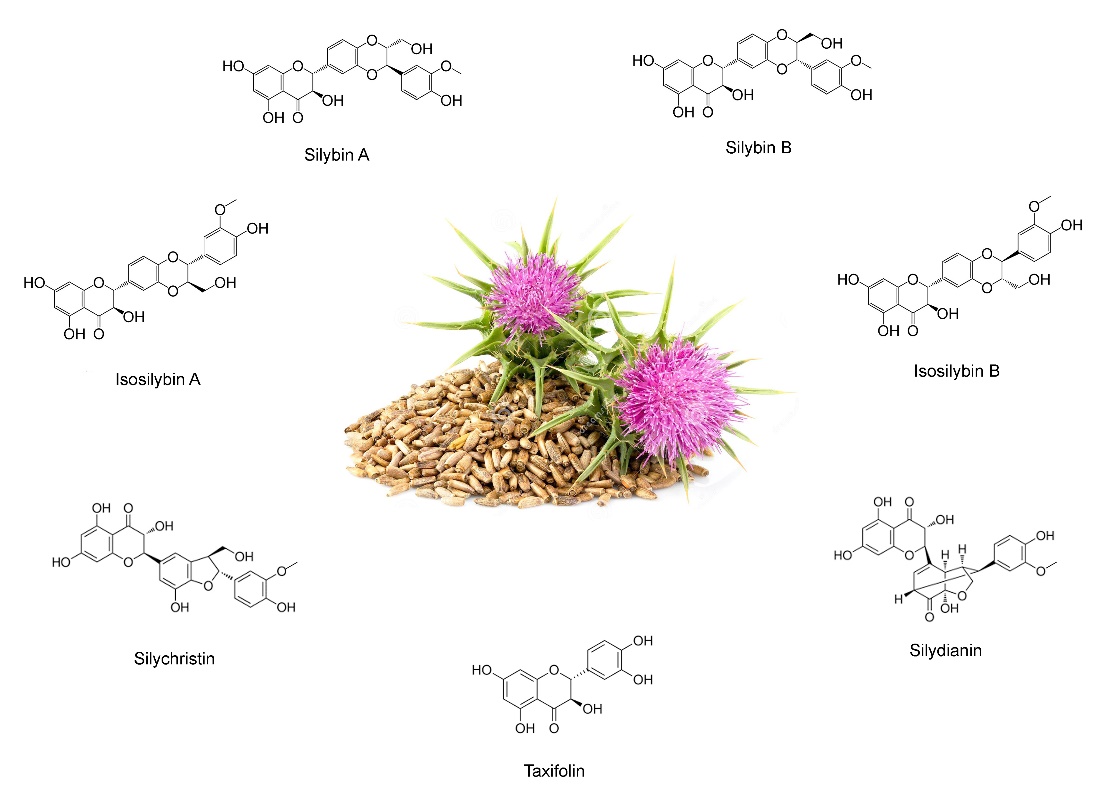


**S1 File** **Fig E.** Chemical structure of main constituents of SIL extract.

**S1 File** **Fig F.** Fluorescence emission spectra of (A) Nano A and (B) Nano B, excited at different wavelengths.

**S1 File** **Fig G.** Cytotoxicity of SIL A, SIL B, and their respective nanoparticles evaluated by MTT-based viability assay.


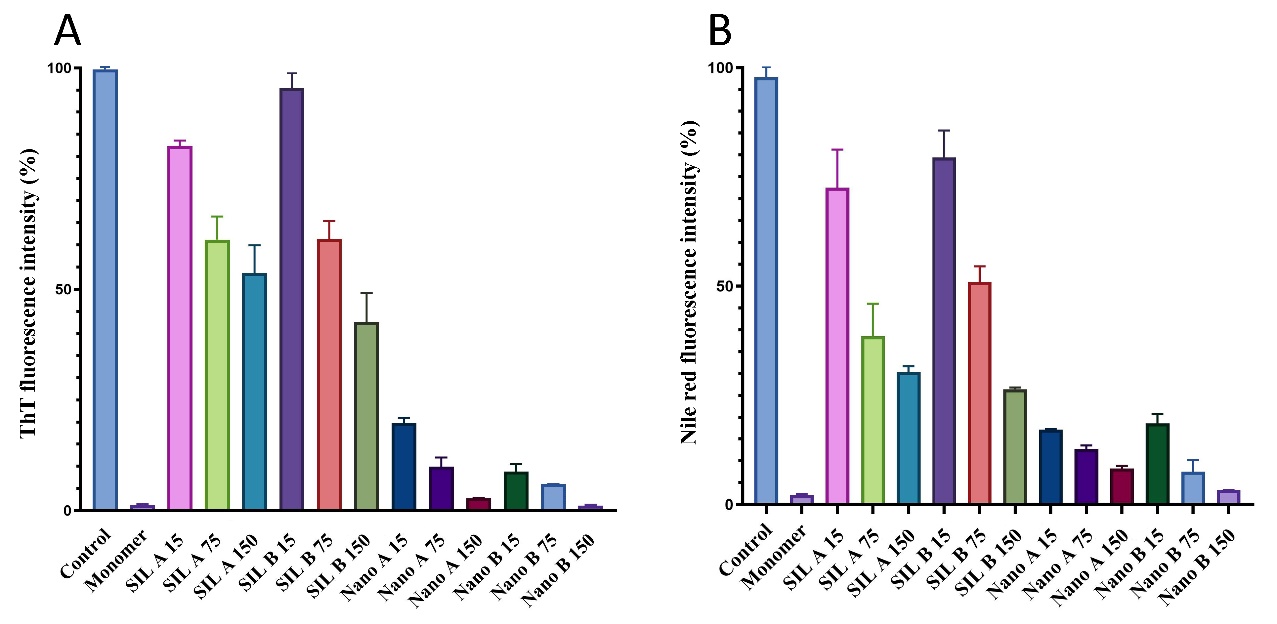


**S1 File** **Fig H.** Quantification of (A) ThT and (B) NR fluorescence intensities of human insulin samples incubated in the presence of various concentrations of SIL A, SIL B, Nano A, and Nano B. The concentrations of compounds are μg/mL. The results are the average of 5 fluorescence images.

**S1 File Fig I.** Congo red (CR) binding absorption spectra of α-syn samples incubated alone or with increasing concentrations of SIL A, SIL B, Nano A, and Nano B. CR absorbance in the presence of PBS and α-syn monomer are also indicated.


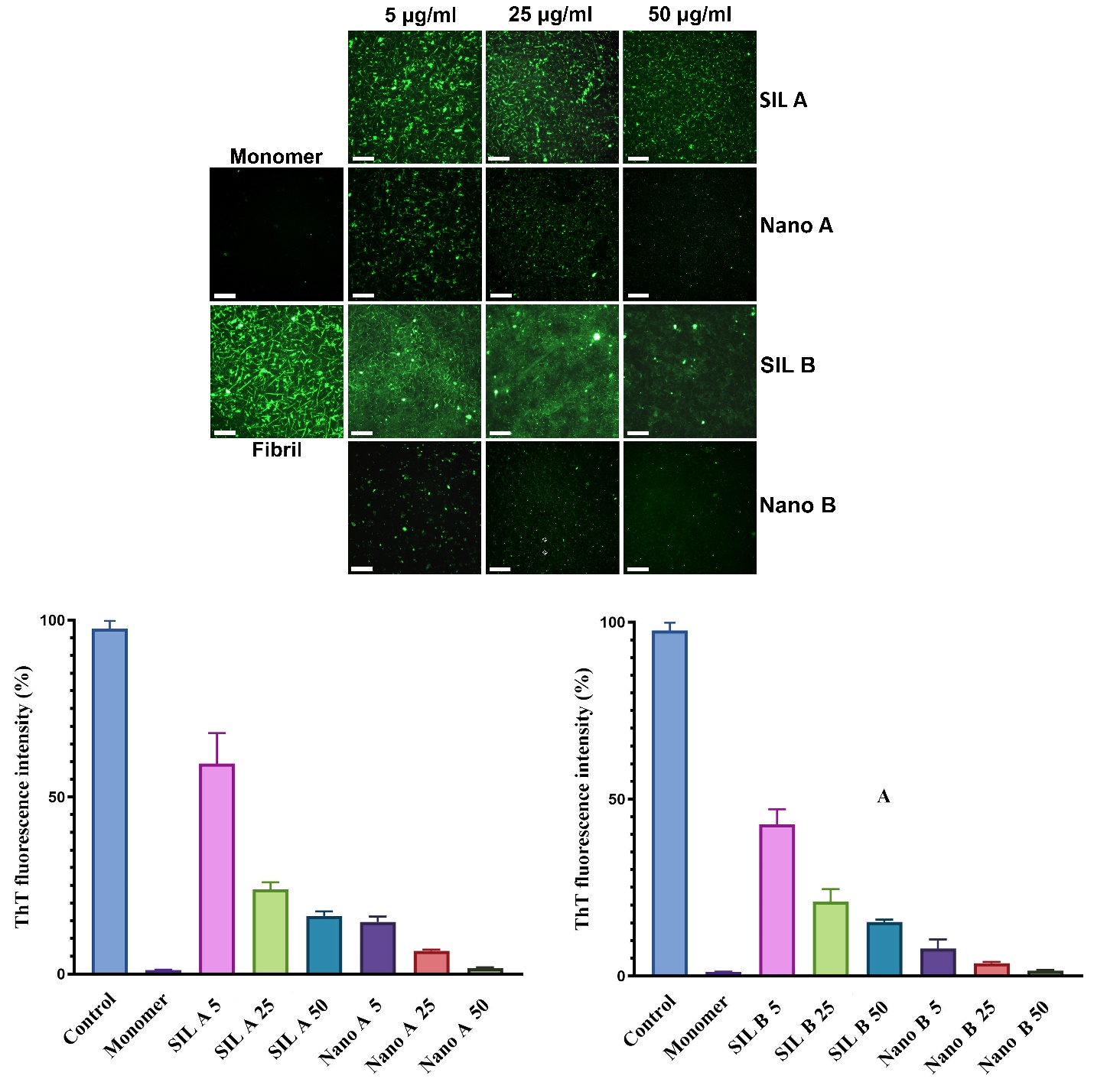


**S1 File** **Fig J.** ThT fluorescence microscopy images of α-syn samples incubated alone or with increasing concentrations of SIL A, SIL B, Nano A, and Nano B for 96 h. Graph shows quantification of ThT fluorescence images. The concentrations of compounds are μg/mL. The results are the average of 5 fluorescence images. The scale bars represent 500 nm.


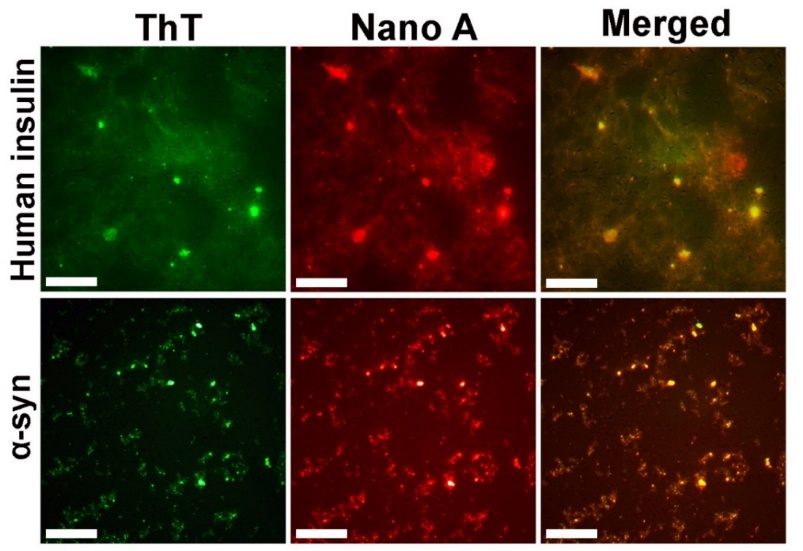


**S1 File Fig K.** Co-staining of human insulin and *α*-syn amyloid aggregates with ThT and Nano A. The left and middle panels indicate green and red fluorescence of amyloid fibrils stained by ThT and Nano A, respectively. The right panels are the merged images of ThT and Nano A indicating co-localization of ThT and Nano A on amyloid fibrils. The scale bar represents 500 nm. The co-staining experiments were done according to our previous report [36].
